# Obicetrapib—the Rebirth of CETP Inhibitors?

**DOI:** 10.1007/s11883-024-01231-5

**Published:** 2024-08-16

**Authors:** Bliss Chang, Luke J. Laffin, Ashish Sarraju, Steven E. Nissen

**Affiliations:** 1 Department of Cardiovascular Medicine, Cleveland Clinic Foundation, Cleveland, OH USA; 2 Section of Preventive Cardiology and Rehabilitation, Department of Cardiovascular Medicine, Cleveland Clinic Foundation, 9500 Euclid Avenue, Mail Code JB1, Cleveland, OH 44195 USA; 3 Cleveland Clinic Coordinating Center for Clinical Research, Cleveland Clinic Foundation, Cleveland, OH USA

**Keywords:** Obicetrapib, CETP, Cholesterylester transfer protein inhibitor, Low-density lipoprotein, Lipid lowering

## Abstract

**Purpose of Review:**

To provide perspective on the current development status, and potential future role, of obicetrapib, a third-generation cholesterylester transfer protein (CETP) inhibitor. Obicetrapib has received recent attention following positive Phase II clinical trial data and initiation of Phase III trials for the treatment of dyslipidemia and atherosclerotic cardiovascular disease (ASCVD).

**Recent Findings:**

The ROSE and ROSE2 trials are Phase II studies that examined the lipid lowering effects of obicetrapib in patients on pre-existing high-intensity statin therapy. Obicetrapib significantly reduced key dyslipidemia biomarkers including low density lipoprotein cholesterol (LDL-C), Apolipoprotein B (Apo B), and non-high-density lipoprotein cholesterol (non-HDL-C) while increasing high-density lipoprotein cholesterol (HDL-C). Four phase III clinical trials, including a cardiovascular outcomes trial, are ongoing.

**Summary:**

Preliminary data for obicetrapib shows favorable effects on dyslipidemia, which could theoretically lead to a decrease in ASCVD clinical events. Short-term safety data in preliminary studies shows no significant safety signals.

## Introduction

Coronary artery disease (CAD) remains the leading cause of mortality globally with a continuous increase in deaths since 2000 despite the availability of many risk reduction measures [1, 2]. Notably, CAD features a strong and direct link between disease-modifying strategies and a single biomarker – plasma low-density lipoprotein cholesterol (LDL-C). After the Nobel Prize-winning discovery of the LDL-C receptor in 1974 by Drs. Joseph Goldstein and Michael Brown, scientists and pharmaceutical companies raced to find an effective medicine to lower LDL-C levels, ultimately leading to the development of statins. High-intensity statin therapy remains the cornerstone for reduction in major adverse cardiac events (MACE), all-cause mortality, and plaque progression in most individuals with coronary disease [3, 4]. However, approximately 50% of eligible patients do not consistently adhere to statins due to hesitancy and concerns about adverse effects [5–7]. Furthermore, a significant proportion of patients fail to achieve an adequate reduction in LDL-C as recommended by US and global guidelines [8–11]. Notably, the 2019 European Society of Cardiology (ESC) dyslipidemia guidelines recommend a target LDL-C level of < 55 mg/dL and a minimum 50% reduction from baseline LDL-C level in high-risk patients, both for primary and secondary prevention [11]. Alternative lipid-lowering therapies approved by the United States Food and Drug Administration (FDA) include proprotein convertase subtilisin/kexin type 9 (PCSK9) monoclonal antibodies and inclisiran, ezetimibe, and bempedoic acid. However, each possesses unique challenges including high-cost, access, tolerability, and patient preference regarding administration of injectable agents.

While LDL-C has been the principal biomarker targeted for lipid-lowering therapy, accumulating data (including Mendelian randomization studies) demonstrate that all Apolipoprotein B-100 (Apo B) containing lipid particles have a significant role in atherogenesis by facilitating the accumulation of lipid-rich particles within the arterial wall leading to inflammation (now understood as a major drivers of atherosclerosis) [12, 13]. Apo B is the major constituent of atherogenic lipid particles including LDL-C and very low-density lipoprotein cholesterol (VLDL-C). Of note, the measurement of non-high-density lipoprotein cholesterol, which includes all Apo B-containing lipid particles including LDL-C, VLDL-C, and lipoprotein (a), predicts MACE better than LDL-C alone [14].

As the contemporary search for effective and well tolerated lipid lowering therapies with favorable effects on LDL-C and Apo B has evolved, a familiar, yet forgotten therapeutic class known as cholesteryl ester transfer protein (CETP) inhibitors have received renewed attention. The newest CETP inhibitor, obicetrapib, is currently under active investigation in multiple clinical trials and shows significant promise as a means to favorably modify lipids and theoretically reduce risk of atherosclerotic cardiovascular disease (ASCVD). The following narrative explores the history of CETP inhibition, including earlier iterations, one of which resulted in increased risk of ASCVD, explains how obicetrapib differs from prior CETP inhibitors, provides an overview of the available data on the effects of obicetrapib on atherogenic biomarkers, and discusses the future of obicetrapib including the ongoing clinical trials. These data provide a framework on how this therapeutic agent may ultimately fit in clinical lipid-lowering treatment paradigms.

## History of CETP Inhibitors

CETP inhibitors reduce transfer of cholesterol from HDL-C particles to LDL-C and VLDL-C, resulting in increased HDL-C levels and reduction in LDL-C and Apo B. The initial realization that CETP inhibition may assist in treatment and prevention of CAD originated from the discovery in 1990 that genetic CETP deficiency led to markedly increased levels of HDL-C and a strong inverse relationship with prevalence of CAD [15]. Pre-clinical studies demonstrated delayed development of atherosclerosis in CETP knock-out studies and showed accelerated atherosclerosis in CETP knock-in models [16]. Several subsequent human studies including the Copenhagen City Heart Study, demonstrated that two common CETP reduced-function polymorphisms were associated with reduced MACE and mortality [17]. Initially, the strong causal relationship between LDL-C and MACE was considered an added benefit of CETP inhibitors beyond the observed increases in HDL-C.

The initial attempts to develop CETP inhibitors were strikingly unsuccessful. In 2006, the first CETP inhibitor, torcetrapib, demonstrated excess mortality in the phase III ILLUMINATE trial which was prematurely terminated at the recommendation of the Data Monitoring Committee (DMC). Understandably, this greatly reduced enthusiasm for other CETP inhibitors, but upon further examination of the torcetrapib data, the excess mortality was associated with off-target drug effects related to this drug’s unique structure rather than CETP inhibition per se [18]. In post-hoc analyses, the increased mortality was attributed predominantly to an increase in aldosterone which was accompanied by an approximately 5 mm Hg increase in systolic blood pressure and hypokalemia. In the context of reduction in events with anti-hypertensive agents, particularly drugs inhibiting the renin–angiotensin–aldosterone system, an increase in events with increased aldosterone levels seemed to explain the toxicity of torcetrapib. Other untoward electrolyte imbalances (potassium, sodium, bicarbonate) were also observed with torcetrapib. Torcetrapib produced a 24.9% decrease in LDL-C after 12 months treatment and increased HDL-C by 72.1% but these benefits did not overcome the drug’s toxicity. The 2007 ILLUSTRATE trial was a large, 1188 patient intravascular ultrasound (IVUS) study that showed no benefits on progression of atherosclerosis, although intriguingly the secondary endpoint demonstrated marginally significant benefits [19].

Subsequent, in 2012, dalcetrapib, a notably weak CETP inhibitor, failed to show a benefit on cardiovascular mortality or MACE in patients with recent acute coronary syndrome in the Phase III placebo-controlled Dal-OUTCOMES trial [20]. A pre-specified interim analysis demonstrated lack of efficacy and the trial was stopped prematurely. Interestingly, in that study, dalcetrapib had no significant effect on LDL-C or Apo B although the drug did raise HDL-C moderately. A more potent CETP inhibitor, evacetrapib, in the phase III ACCELERATE trial, also failed to reduce MACE in high-risk secondary prevention patients despite an apparent 31% reduction in LDL-C and a 133% increase in HDL-C. The trial was terminated prematurely for futility at the recommendation of the DMC after a median follow-up of 26 months [21]. However, the apparent 31% reduction in LDL-C was artifactually greater in magnitude than would likely have been seen with more accurate assays such as beta-quantification.

While there are several methods for measuring LDL-C levels, beta-quantification is considered the reference measurement procedure [22]. Beta-quantification involves ultracentrifugation to physically separate out the VLDL-C and chylomicron components prior to measuring the remaining LDL-C and HDL-C. Indirect LDL-C measurement using the traditional Friedewald equation underestimates the true LDL-C level which may falsely exaggerate the effectiveness of CETP inhibitors in reducing LDL-C. The key issue with LDL-C measurement via the Friedewald equation is the incorporation of triglycerides which estimates VLDL-C despite a significant variability in the ratio of cholesterol to triglycerides in VLDL-C [23].

In 2017, outcomes for anacetrapib were reported in the randomized, controlled REVEAL trial, a > 30,000 participant study in secondary prevention patients on high intensity statin therapy. In a much-needed “win” for CETP inhibitors, anacetrapib reduced the primary composite outcome of coronary death, myocardial infarction, or coronary revascularization (rate ratio, 0.91; 95% confidence interval, 0.85 to 0.97; P = 0.004) but not overall mortality or cardiovascular death [24]. The baseline mean LDL-C level in that study was very low, 61 mg/dL. The study showed a reduction of up to 40% for LDL-C and 18% for non-HDL cholesterol, while raising HDL-C by 102%. Notably, in the REVEAL trial, anacetrapib had a much more modest LDL-C lowering effect (17% versus 41% reduction) when calculated via the beta-quantification approach. [24]. There was also a signal for a reduction in the incidence of new-onset diabetes mellitus in patients randomized to anacetrapib [24]. However, the pharmaceutical company developing anacetrapib (Merck & Co., Inc.) ceased further development of the drug, presumably deeming the CV risk reduction observed with anacetrapib insufficient to pursue regulatory approval.

## How Obicetrapib is Different

Obicetrapib is an extremely potent CETP inhibitor with a much larger effect on atherogenic lipoproteins. After the failure to commercialize the first four CETP inhibitors, the ROSE study reported much larger LDL-C lowering with obicetrapib, the fifth commercially developed CETP inhibitor [25]. In this phase II study, 120 participants were randomized to 8 weeks of treatment with placebo or either 5 mg or 10 mg of obicetrapib. All enrolled patients were treated with maximally tolerated high-intensity statin therapy for a minimum of eight weeks. The mean baseline pre-treatment LDL-C ranged from 88 to 95 mg/dL. Obicetrapib treatment, compared with placebo, produced a mean LDL-C decrease of 51% with the 10 mg dose and the 42% with 5 mg dose measured using the preferred beta quantification method. Additionally, lipoprotein(a) [Lp(a)] was reduced in a dose-dependent fashion by 33.8% and 56.5% for obicetrapib 5 mg and 10 mg, respectively, whereas ApoB was reduced by 24.4% and 29.8%. Non-HDL-C decreased 44% with the higher dose and HDL-C increased by 165%. There was no signal for significant differences in adverse events or other off-target effects for obicetrapib in this small, short-term study, providing preliminary evidence of the potential safety of using this higher efficacy CETP inhibitor.

ROSE2, a phase II clinical trial, demonstrated that the combination of obicetrapib and ezetimibe provided greater lipid lowering compared with obicetrapib alone or ezetimibe alone [26]. Combination therapy for 12 weeks lowered LDL-C by 63.4% compared with a 43.5% reduction for obicetrapib monotherapy. This magnitude of reduction in atherogenic lipoproteins is comparable to the addition of a PCSK9 inhibitor to a high intensity-statin [27]. In this trial, 87.1% of patients on combination therapy compared with 42.3% receiving obicetrapib monotherapy reached the most stringent LDL-C goal of < 55 mg/dL. There were also favorable signals for lowering Lp(a) and the more atherogenic small dense LDL-C.

**Fig. 1:**
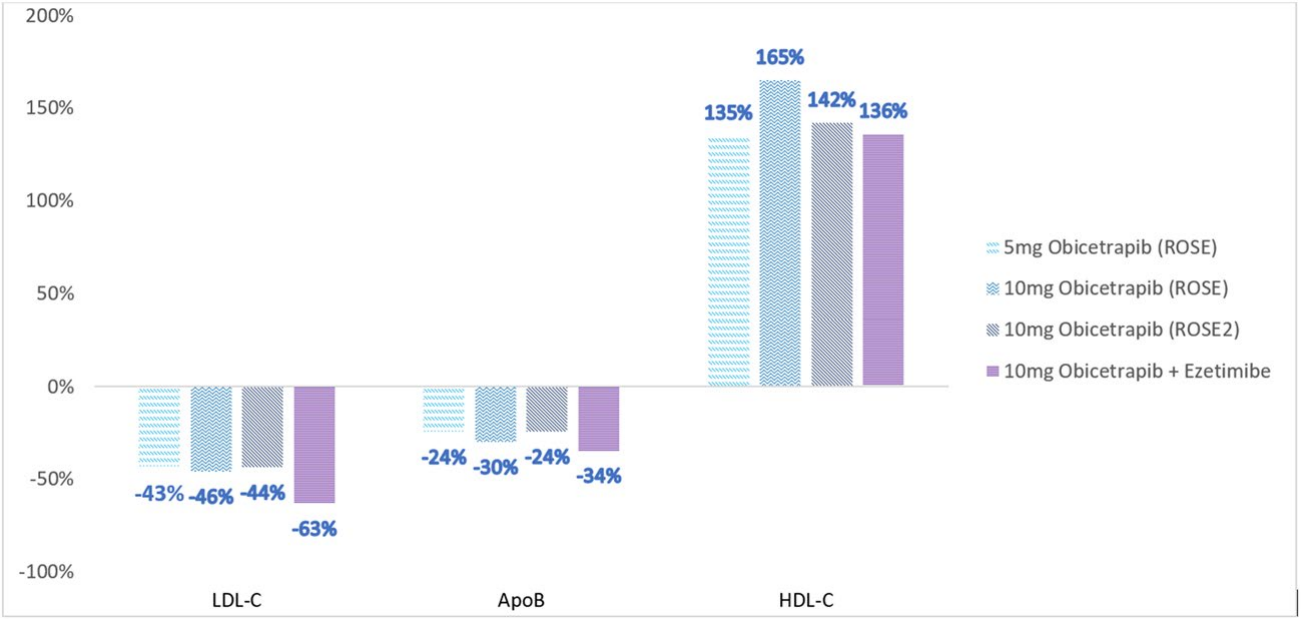
Changes in Key Lipid Markers in Phase II Obicetrapib Trials [22, 24]

The ROSE studies provide renewed enthusiasm for further investigation of highly potent CETP inhibitors such as obicetrapib. The LDL-C lowering efficacy of obicetrapib was demonstrated in patients already on high-intensity statin therapy, which replicates the likely clinical use of non-statin therapies in the contemporary era of lipid lowering. Figure 1 summarizes the dose-dependent reductions in key lipid biomarkers in the phase II trials of obicetrapib including Apo B.

## Potential Harms of CETP Inhibition

A Danish study in 2021 analyzing the long-term benefits and harms of patients genetically deficient in CETP found a higher risk of age-related macular degeneration [28]. While the mechanism is unclear, these data raise a potential concern: is the potent inhibition of CETP safe? There is epidemiological evidence that very high HDL-C levels (loosely defined as at least > 80 mg/dL) are associated with adverse outcomes including increased cardiovascular mortality and risk of advanced age-related macular degeneration particularly in those of Asian and European ancestry [29, 30]. However, the long-term benefits and risks of potent CETP inhibition using obicetrapib will soon be available to definitively answer this question.

## Future Directions

Despite the difficulties encountered by the first four CETP inhibitors, there is reason to be cautiously optimistic with regards to the ongoing development of the fifth CETP inhibitor, obicetrapib, based on the ROSE and ROSE2 results. There is now a large, multinational, placebo-controlled, randomized cardiovascular outcomes trial underway, PREVAIL (NCT05202509) which is studying 10 mg of obicetrapib compared with placebo in patients with pre-established atherosclerotic cardiovascular disease (ASCVD) [31]. This phase III study sponsored by New Amsterdam Pharmaceuticals and led by Monash University in Australia completed enrollment of 9,541 patients in 2022 and has an anticipated completion date of late 2026. The primary outcome is the effect of obicetrapib on MACE in patients treated with maximally tolerated lipid lowering therapy.

There are three additional ongoing phase III trials (Table 1) are investigating obicetrapib in varying patient populations and medication combinations. These include BROOKLYN, BROADWAY, and TANDEM. The BROOKLYN trial (NCT05425745) is a 354 participant, placebo-controlled, double-blind, randomized trial, which began in 2022, investigating the LDL-C lowering efficacy of obicetrapib in patients with heterozygous familial hypercholesterolemia receiving maximally tolerated lipid lowering therapy but not achieving a target LDL-C levels < 70 mg/dL [32]. Because heterozygous familial hypercholesterolemia remains largely undiagnosed, the public health effects of this trial will hopefully increase awareness of familial hypercholesterolemia.

**Table 1:** Ongoing Phase III Randomized Control Trials for Obicetrapib

Trial	Treatment Population	Primary Outcome	Projected # of Participants	Pre-existing Lipid -Lowering Therapy	Start Date	Projected Completion Date
PREVAIL (NCT05202509)	Prior ASCVD	MACE	9,541	Yes	February 2022	Late 2026
BROOKLYN (NCT05425745)	Heterozygous FH not meeting LDL target	Change in LDL-C at 12 Weeks	300	Yes	July 2022	Q3, 2024
BROADWAY (NCT05142722)	Heterozygous FH or prior ASCVD not meeting LDL-C target	Change in LDL-C at 12 Weeks	2,532	Yes	December 2021	Q3, 2024
TANDEM (NCT06005597)	Heterozygous FH or prior ASCVD or high risk for ASCVD	Change in LDL-C at 12 Weeks	400	Yes	March 2024	November 2024

Abbreviations: *ASCVD* atherosclerotic cardiovascular disease, *FH* familial hypercholesterolemia, *LDL-C* low-density lipoprotein cholesterol, *MACE* Major adverse cardiovascular events

BROADWAY (NCT05142722) is a much larger phase III placebo-controlled, randomized trial of 2,532 participants evaluating the LDL-C lowering efficacy of obicetrapib in individuals with either heterozygous familial hypercholesterolemia and/or a prior ASCVD and a LDL-C ≥ 70 mg/dL) [33]. BROADWAY is estimated to complete before the end of 2024.

Finally, TANDEM (NCT06005597) is a 400-participant placebo-controlled, randomized trial designed to evaluate the LDL-C lowering efficacy of fixed-dose combination therapy of obicetrapib and ezetimibe in patients who have heterozygous familial hypercholesterolemia, prior ASCVD, or are high risk for ASCVD [34]. This is a 2 × 2 factorial trial in participants on maximally tolerated lipid lowering therapy at the time of enrollment. The treatment arms include 10 mg obicetrapib, 10 mg obicetrapib in combination with 10 mg ezetimibe, 10 mg ezetimibe, or placebo.

## Conclusion

Phase II studies of obicetrapib demonstrate favorable effects on atherogenic lipid biomarkers including LDL-C, Apo B, and non-HDL-C levels. If phase III trial data continue to support the efficacy and safety of obicetrapib for LDL-C lowering as monotherapy or as a fixed-dose combination with ezetimibe, this CETP inhibitor has the potential to become a viable lipid-lowering agent in patients receiving maximally tolerated statin therapy but not a goal LDL-C levels. Although ezetimibe is the usual second-line agent in contemporary lipid lowering treatment, the degree of LDL-C and Apo B reduction achieved with the addition of obicetrapib is much greater than observed with the addition of ezetimibe (typically 16–18% reduction in LDL-C). PCSK9 inhibitors are effective, but use is often limited by cost or availability in many regions of the world. Some patients dislike self-injection of the PCSK9 monoclonal antibodies every 2 weeks, although the short interfering RNA, inclisiran, can be administered twice yearly in a physician’s office which some patients prefer. Obicetrapib has the convenience of oral administration with an additional potential benefit, reduction in Lp(a) concentrations that cannot be reduced effectively with existing therapies. If reduction in Lp(a) demonstrates outcome benefits in current trials of nucleic acid-based therapies, obicetrapib might represent an alternative treatment for some patients.

While we have emerging evidence of the lipid-lowering efficacy of obicetrapib, cardiovascular outcome data, such as those from the ongoing PREVAIL study, are crucial in defining obicetrapib’s role in future clinical care. Characterization of the long-term safety of CETP inhibition will be required for regulatory approval, which will be provided by the BROOKLYN and BROADWAY trials. Obicetrapib with multiple ongoing trials may be the one final opportunity for CETP inhibitors to make a comeback as a component of contemporary ASCVD prevention and treatment.

## Data Availability

No datasets were generated or analysed during the current study.
